# Aging Increases Cross-Modal Distraction by Unexpected Sounds: Controlling for Response Speed

**DOI:** 10.3389/fnagi.2021.733388

**Published:** 2021-09-15

**Authors:** Alicia Leiva, Pilar Andrés, Fabrice B. R. Parmentier

**Affiliations:** ^1^Neuropsychology & Cognition Group, Department of Psychology & Research Institute of Health Sciences (iUNICS), University of the Balearic Islands, Palma, Spain; ^2^Balearic Islands Health Research Institute (IdISBa), Palma, Spain; ^3^Department of Psychology, University of Western Australia, Perth, WA, Australia

**Keywords:** deviance distraction, aging, auditory distraction, cross-modal attention, oddball, attention capture

## Abstract

It is well-established that task-irrelevant sounds deviating from an otherwise predictable auditory sequence capture attention and disrupt ongoing performance by delaying responses in the ongoing task. In visual tasks, larger distraction by unexpected sounds (deviance distraction) has been reported in older than in young adults. However, past studies based this conclusion on the comparisons of absolute response times (RT) and did not control for the general slowing typically observed in older adults. Hence, it remains unclear whether this difference in deviance distraction between the two age groups reflects a genuine effect of aging or a proportional effect of similar size in both groups. We addressed this issue by using a proportional measure of distraction (PMD) to reanalyze the data from four past studies and used Bayesian estimation to generate credible estimates of the age-related difference in deviance distraction and its effect size. The results were unambiguous: older adults exhibited greater deviance distraction than young adults when controlling for baseline response speed (in each individual study and in the combined data set). Bayesian estimation revealed a proportional lengthening of RT by unexpected sounds that was about twice as large in older than in young adults (corresponding to a large statistical effect size). A similar analysis was carried out on the proportion of correct responses (PC) and produced converging results. Finally, an additional Bayesian analysis comparing data from cross-modal and uni-modal studies confirmed the selective effect of aging on distraction in the first and not the second. Overall, our study shows that older adults performing a visual categorization task do exhibit greater distraction by unexpected sounds than young adults and that this effect is not explicable by age-related general slowing.

## Introduction

The ability to filter out task-irrelevant stimuli to concentrate on a task at hand plays a fundamental role in efficient functioning. Yet, being entirely oblivious to task-irrelevant stimuli can be counter-productive. For example, the reader of this article may be using selective attention to help process the meaning of this text while filtering out nearby conversations, the noise of a passing car, or other extraneous stimuli. However, should the fire alarm suddenly go off, detecting such unexpected auditory stimulus would be of paramount importance to interrupt ongoing activities (reading) and modifying one’s behavior in the service of a new goal (exiting the building). From an attentional perspective, this example illustrates the balance between selective attention (filtering out irrelevant stimuli) and change detection (allowing unexpected stimuli to break through attentional filters) mechanisms. Abundant research has evidenced the existence and neurological underpinnings of mechanisms capable of detecting the occurrence of stimuli violating predictions and capturing our attention in an obligatory manner (Schröger, 1996, 2007; Escera et al., 1998; Schröger and Wolff, 1998; Winkler, 2007; Wessel and Aron, 2013; Parmentier, 2014). While adaptive, such mechanisms present one downside: the interruption of ongoing cognitive activities yields a transient reduction in ongoing task performance which, when attention-capturing stimuli are truly irrelevant, amounts to distraction.

One class of stimuli repeatedly shown to grab attention are sudden changes (oddball stimuli) in a sequence of otherwise repeated or predictable (standard) sounds (see Parmentier, 2014, for a review). At the center of the present study is the finding that distraction by unexpected sounds is significantly greater in older than in young adults when the task at hand is visual (Andrés et al., 2006; Parmentier and Andrés, 2010; Leiva et al., 2015b, 2016), and not when it is auditory (Leiva et al., 2015a, b). Here, our primary aim was to re-evaluate the cross-modal studies to examine an issue prominent in the field of aging research, but which has been overlooked in past studies on aging and distraction by unexpected sounds: the role of baseline response speed. We used Bayesian statistics to re-analyze the data from four past studies and establish whether their conclusions remain valid when factoring out age-related variations in response speed. As a secondary aim, for completeness, we also performed a similar analysis on response accuracy. Finally, we contrasted these data with those from two past studies using uni-modal oddball tasks and confirmed that aging selectively affect distraction in the cross-modal oddball task.

### Distraction by Unexpected Sounds

Task-irrelevant sounds unexpectedly deviating from a predictable sequence induce an orienting response characterized by specific electrophysiological responses and behavioral distraction (Schröger, 1996, 2005; Escera et al., 1998; Berti and Schröger, 2001; Horváth et al., 2008; Parmentier et al., 2008; Berti, 2013; Parmentier, 2014; Rosburg et al., 2018). This is typically studied in simple forced-choice categorization tasks involving task-irrelevant sounds (Escera et al., 1998; Pacheco-Unguetti and Parmentier, 2014; Parmentier et al., 2014, 2018), though it is not specific to that task and has been observed in others such as duration judgment, go-no go, visual matching, serial recall, or gap detection tasks (Berti and Schröger, 2001; Hughes et al., 2005; Bendixen et al., 2010; Li et al., 2013; Röer et al., 2015, 2017, 2018; Pacheco-Unguetti and Parmentier, 2016; Körner et al., 2017; Vachon et al., 2017; Volosin et al., 2017; Volosin and Horváth, 2020).

In cross-modal oddball tasks, target stimuli are typically visual, each preceded by a task-irrelevant sound which is repeated on most trials and unexpectedly replaced by a different sound on some trials. These unexpected changes typically entail longer response times (RT) and sometimes a small reduction in response accuracy (see Parmentier, 2014, for a review). This effect is thought to reflect the transient inhibition of motor actions (Wessel and Aron, 2013; Parmentier, 2016; Wessel, 2017; Vasilev et al., 2019, 2021; Wessel and Huber, 2019) and the involuntary shift of attention to and from the unexpected sound (Schröger, 1996; Parmentier et al., 2008). These behavioral manifestations are thought to reflect an adaptive interruption of ongoing actions, the assessment of whether a new action plan must be selected and, if not, the reactivation of the relevant task set (Corbetta and Shulman, 2002; Verbruggen et al., 2014; Wessel and Aron, 2017). Importantly, unexpected sounds distract because they violate the cognitive system’s predictions, not because they are rare *per se* (Schröger et al., 2007; Parmentier et al., 2011; Volosin and Horváth, 2014; Schröger and Roeber, 2021). Consequently, distraction lessens or disappears when unexpected sounds are made predictable (e.g., Sussman et al., 2003; Horváth and Bendixen, 2012; Parmentier and Hebrero, 2013), but it appears immune to the predictability of our own behavior (Parmentier and Gallego, 2020).

### Aging and Distraction by Unexpected Sounds

Several studies have examined distraction by unexpected sounds in young and older adults. In studies using uni-modal tasks, aging does not appear to modulate deviance distraction. While one study using an auditory duration discrimination task reported an age-related increase in distraction by rare and unexpected changes in pitch (Berti et al., 2013), a subsequent study using a larger sample found no difference between the two age groups (Leiva et al., 2015a). Using a similar task in children, young and older adults, Horváth et al. (2009) found no difference between young and older adults with respect to behavioral deviance distraction. Similar degrees of behavioral distraction were also observed in young and high-performing older adults (Getzmann et al., 2013).

In contrast to uni-modal studies, cross-modal oddball studies consistently show an age-related increase in distraction by unexpected sounds. Using a visual digit categorization task, Andrés et al. (2006); see Parmentier and Andrés, 2010, for a replication) reported a twofold increase in distraction in older compared to young adults as measured from RT. A study comparing the two age groups in uni-modal and cross-modal oddball tasks confirmed a selective effect of aging on distraction in the cross-modal task (Leiva et al., 2015b). Converging findings were also reported in a cross-sectional study comparing children, young and older adults with respect to deviance distraction, working memory capacity and response inhibition (Leiva et al., 2016). The exact origin of the selective effect of aging on deviance distraction in the cross-modal as opposed to uni-modal tasks has not clearly been identified yet. The cross-modal task differs from the uni-modal in that it involves attention crossing sensory boundaries, which has been hypothesized as a one contributor to the effect (Parmentier et al., 2008). Hence, one reasonable hypothesis is that aging selectively affects the deployment of attention across sensory modalities while within-modality shifts are relatively unscathed (Leiva et al., 2015b).

### The Present Study: Controlling for Age-Related Differences in Response Speed

Evidence shows that, relative to a repeated and predictable sound, unexpected sounds distract participants from the task at hand and yield longer RT. Interestingly, when this task is visual, distraction is significantly larger in older than in young adults (Andrés et al., 2006; Parmentier and Andrés, 2010; Leiva et al., 2015b, 2016). However, one factor has been overlooked in these studies: the general slowing exhibited by older adults. Indeed, while these studies reported age-related differences in RT between unexpected and standard sound conditions, the baseline response speed of participants was not considered. In other words, one cannot rule out the possibility that this age-related increase of distraction may simply be a proportional effect.

It is well-established that variations in cognitive and response speed constitute a key general factor in accounting for age-related changes in memory and cognition (Shimamura, 1994). Gradual slowing is a well-documented hallmark of aging (Cerella, 1985; Rogers and Fisk, 1990; Salthouse and Meinz, 1995; Salthouse, 1996, 2000). Not surprisingly, older adults were overall slower than young adults in all four cross-modal studies reviewed above. Hence, before concluding that unexpected sounds selectively affect older adults in cross-modal oddball tasks, it is necessary to examine whether the proportional increase in RT in the unexpected sound condition relative to the standard condition is greater for older than for young adults.

Here, we reanalyzed the data from the four cross-modal oddball studies on aging (Andrés et al., 2006; Parmentier and Andrés, 2010; Leiva et al., 2015b, 2016) to test for an age-related increase in distraction while controlling for baseline response speed. To examine this issue, we compared young and older adults using a proportional metric of distraction whereby distraction (RT unexpected – RT standard) was pitched against baseline response speed (RT standard). We then used Bayesian estimation on the combined data set to derive point-estimates and 95% high density intervals (95% HDI) of the mean difference in proportional distraction and its effect size. The hypotheses were simple: If the age-related difference in RTs between unexpected and standard sound trials reflect a genuine increase in distraction, then (1) we should find significant differences in proportional distraction effects between the two age groups and (2) Bayesian estimation should reveal credible value ranges for the age group difference in proportional distraction and its effect size that exclude zero. In contrast, if the age-related increase in distraction reported in previous studies is a mere reflection of a proportional slowing of responses, then (1) we should find no difference between the two age groups when controlling for baseline response speed, and (2) Bayesian estimation should highlight zero as a credible value for the mean age-related difference in distraction and its effect size.

## Methods

We present here a brief description of the key methodological aspects of the four cross-modal oddball studies (Andrés et al., 2006; Parmentier and Andrés, 2010; Leiva et al., 2015b, 2016) relevant to our purpose. Our focus was on the comparison of the RT and response accuracy of young and older adults in oddball tasks in which standard and unexpected sounds were presented while participants performed a visual categorization task. Hence, from the study by Leiva et al. (2015b), we used data from the cross-modal condition, not the uni-modal condition. From the study of Andrés et al. (2006), we used the data from the sound blocks, not the silent blocks. From the study by Leiva et al. (2016), we selected the data from young and older adults, not that of children, in the cross-modal oddball task (not the working memory or response inhibition tasks). In all cases, the first standard trial after an unexpected sound trial was excluded from the analysis, for past work demonstrated that this trial is subject to residual distraction (Ahveninen et al., 2000; Roeber et al., 2003; Berti, 2008).

### Participants

The sample included a total of 204 participants (148 females and 56 males) forming two age groups: 108 young adults (*M* age = 21.62, *SD* = 3.68) and 96 older adults (*M* age = 67.11, *SD* = 8.38). We present in Table 1 (Panel A) a descriptive breakdown of age and sex per study. For an average effect size of *d* = 0.963 (mean of the *d* values for the effect of aging on deviance distraction in the four studies we are revisiting), the statistical power of a *t*-test for this sample size, a Type I error of 0.05, and a one-tailed hypothesis, the statistical power of our study was >0.999. Put differently, given our sample size, the minimum effect size affording a power of 0.95 was *d* = 0.463.

**TABLE 1:** **(A)** Demographic characteristics of the samples from the four studies we reanalyzed. **(B)** Key methodological characteristics of the tasks used in the four studies we reanalyzed.

A						

	Older			Young		
	*n*	F/M	*M* age (SD)	*n*	F/M	*M* age (SD)
**Participants characteristics**						
Andrés et al., 2006	22	6/16	67.95 (8.88)	22	7/15	22.26 (3.73)
Parmentier and Andrés, 2010	20	6/14	68.6 (9.05)	20	8/12	21.75 (3.28)
Leiva et al., 2015b	22	7/15	63.86 (5.61)	22	3/19	21.64 (4.51)
Leiva et al., 2016	32	8/24	64.17 (5.58)	44	11/33	20.66 (3.67)
Overall	96	69/27	67.11 (8.38)	108	79/29	21.62 (3.68)

B									

				Type of sound					
Study	Task	N test trials	Proportion unexpected sound	Standard	Unexpected	Sounds’ duration (ms)	Sound to target interval (ms)	Target duration (ms)	Trial to trial interval (ms)
**Tasks characteristics**									
Andrés et al., 2006	Visual digit categorization	1,200	0.1	600 Hz sinewave tone	60 environmental sounds	200	100	200	1,500
Parmentier and Andrés, 2010	Visual digit categorization	1,200	0.1	600 Hz sinewave tone	60 environmental sounds	200	100	200	1,500
Leiva et al., 2015b	Visual digit categorization	1,440	0.2	600 Hz sinewave tone	White noise	150	150	400	2,200
Leiva et al., 2016	Left/right categorization of cartoon dog	480	0.3	600 Hz sinewave tone	White noise	150	100	200	2,950

### Stimuli and Procedure

The cross-modal oddball task presented the same general structure in all four studies, though with small variations with respect to the exact stimuli, proportion of unexpected sound trials, number of test trials, duration of stimuli, inter-stimuli and inter-trial intervals. For clarity, we present a descriptive comparison of the task characteristics in Table 1 (Panel B). In all cases, the participants’ task was to perform a binary categorization of a visually presented stimulus (a digit in three studies, a cartoon dog in one). A task-irrelevant sound preceded each visual target. The duration of the sounds, sound-to-target, target, and trial-to-trial intervals was fixed within each study and varied slightly across studies. In the majority trials (70–90%, depending on the study), a 600 Hz sinewave tone served as the standard sound while unexpected sounds (white noise or environmental sounds) was used in the remaining trials. Participants were instructed to attend the visual task and endeavor to respond as quickly as possible while trying to make no error. All participants were tested individually in a sound attenuated laboratory. Sounds were presented through headphones with a level of approximately 75 dB SPL.

## Data Analysis

The analysis was twofold. First, to test for the age-related variation in distraction while controlling for performance in the standard condition, we reanalyzed the data from four studies (Andrés et al., 2006; Parmentier and Andrés, 2010; Leiva et al., 2015b, 2016) using a proportional measure of distraction (PMD). While our primary interest lied in the analysis of RT, we also implemented this metric for the proportion of correct responses (PC) for completeness. This measure simply consisted in calculating the ratio between distraction (difference between the standard and unexpected conditions) and performance in the standard condition such that positive PMD values can be interpreted as a proportional detrimental impact of unexpected sounds on performance:


$$PMD_{RT}=\frac{\left(RT_{unexpected}-RT_{standard}\right)}{RT_{standard}}$$

$$PMD_{PC}=\frac{\left(PC_{standard}-PC_{unexpected}\right)}{PC_{standard}}$$

Since Shapiro-Wilk and Levene tests carried out on PMD for RT and proportions of correct responses in each study revealed numerous deviations from normality (which were not solved by log-transforming the data) and homoscedasticity, we used the non-parametric Mann-Whitney’s statistic. Effect size is reported as the rank-biserial correlation (*r*_*B*_). For each comparison, we also report the Bayes Factor (*BF*_10_) to assess the credibility of the experimental hypothesis relative to that of the null hypothesis given the data. Values below 1/3 or >3 are regarded as moderate or substantial support for the null and experimental hypotheses, respectively, while values below 1/10 and 10 are regarded as strong evidence (Jeffreys, 1961; Lee and Wagenmakers, 2013; Dienes, 2014; Quintana and Williams, 2018). Bayes factors were computed using 1,000 iterations. The analysis was conducted using JASP (JASP Team, 2019), using the default normal prior (mean of zero, standard deviation of 0.707). In these analyses, the *a priori* experimental hypothesis was that distraction is greater in older than in young adults. For confirmation and examination of the coherence of the pattern of results across the four studies, we also conducted a 4 (Study) × 2 (Age Group) Bayesian Analysis of variance (BANOVA) on PMD_*PC*_ and PMD_*RT*_. Plots were created using the ggstatsplot (Patil, 2021) and ggplot2 (Wickham, 2016) packages in R (R Core Team, 2021).

Next, we analyzed the complete set of data using Bayesian estimation (Kruschke, 2013, 2015) to provide credible parameters estimates about the difference in PMD between young and older adults (Kruschke, 2013, 2015). This analysis was carried in R (R Core Team, 2021) using the BEST package (Kruschke and Meredith, 2018). Bayesian estimation takes a different approach to null hypothesis significance testing by calculating credible ranges of values for model parameters in the light of the empirical data (Kruschke, 2013; Kruschke and Liddell, 2018). The two key posterior parameters of interest were the point-estimate for the mean difference in PMD between the age groups and its effect size. For completeness, we also report the other posterior parameter (estimated standard deviation and normality of the difference). We used BEST’s minimally informative default priors (“so that the prior has minimal influence on the estimation, and the data dominate the Bayesian inference,” Kruschke, 2013 p. 576). Bayesian estimation reallocates credibility to the model’s parameter in a way that best accommodates the empirical data. The posterior distribution’s parameters were approximated using the Markov Chain Monte Carlo (MCMC) method, which generates a large sample of credible parameter values from the posterior distribution (using BEST’s default size for this sample, or chain length, of 100,000). Credible intervals were calculated in the form of 95% HDI.

## Results

### Re-analysis of Past Studies Using PMD

**FIGURE 1:**
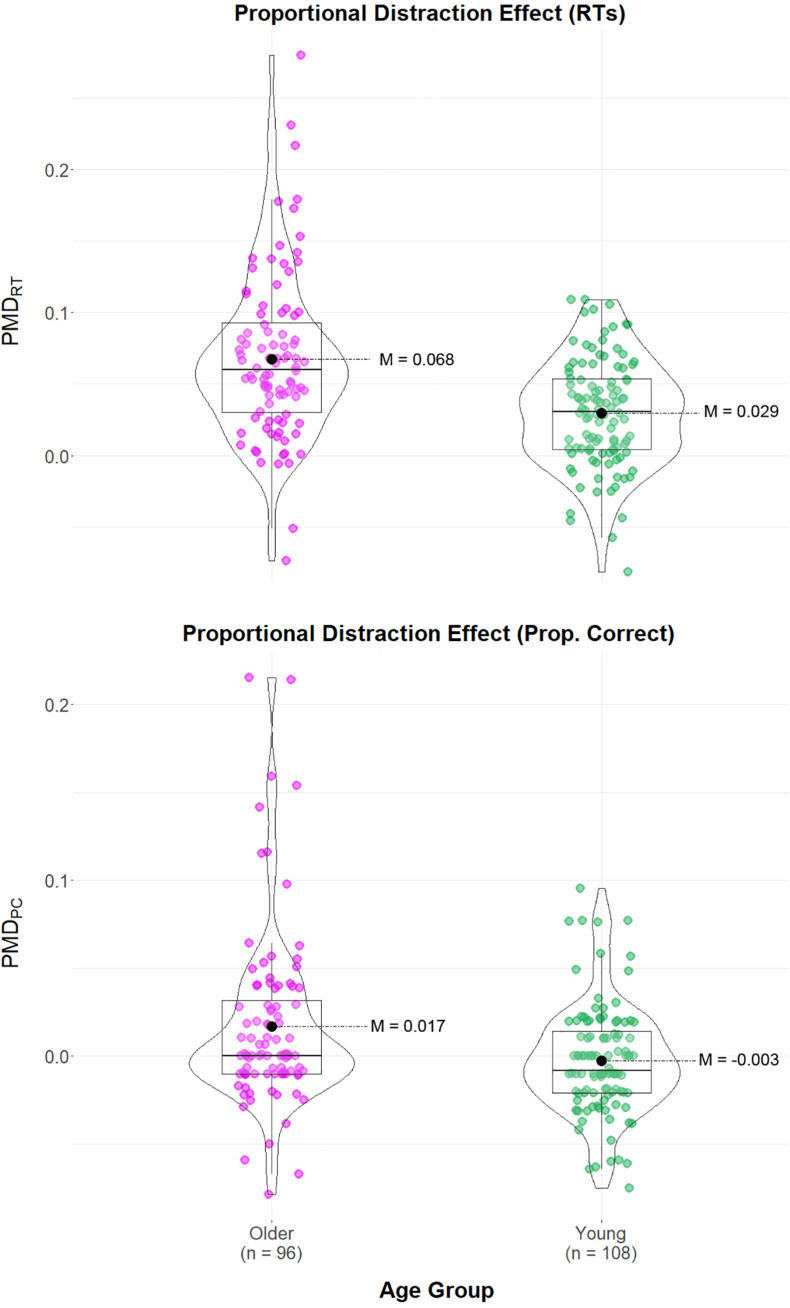
Violin plots of the proportional distraction effect for young and older adults. Colored dots represent individual data point. Black dots indicate group means.

For clarity, we present the full list of statistical results for our first analysis in Table 2 (Panel A). As visible from these results, both non-parametric and Bayesian statistics supported our experimental hypothesis. Deviance distraction was significantly and credibly greater in older adults in all four studies for RT, and in two studies for the PC (while in the remaining two, the results were inconclusive). As visible in Table 2 (Panel B), the BANOVAs carried out on the combined sets of data provided strong (PMD_*PC*_) and extreme (PMD_*RT*_) evidence of the effect of aging on our proportional measures of distraction. While the evidence favored the conclusion that PMD_*PC*_ did not vary across studies, the *BF*_10_ of 0.346 was inconclusive. In contrast, extreme evidence supported the absence of variation of PMD_*RT*_ across studies (*BF*_10_ = 0.003). Importantly, the results supported the absence of interaction between Study and Age Group: *BF*_10_ = 1.243/7.371 = 0.169, and *BF*_10_ = 7308.905/48004.861 = 0.152, for PMD_*PC*_ and PDM_*RT*_, respectively. The PMD_*PC*_ and PMD_*RT*_ values for participants in each age group are presented in Figure 1. The results can be summarized as follows. The strength of the evidence of the impact of aging on PMD_*PC*_ varied across individual studies but was strong in the combined data set. More importantly, the data clearly supported the hypothesis that aging increases PMD_*RT*_, with moderate to strong evidence in individual studies, and extreme evidence in the combined data set.

**TABLE 2:** **(A)** Statistical effect of aging on distraction in each study (asterisks highlight significant effects). **(B)** Results of the Bayesian ANOVAs on response accuracy and response times.

A								

Study	M young (SD)	M older (SD)	Older - Young	*W*	*p*	*r*_*B*_	95% CI r_B_	*BF*_10_
PMD_PC_								
Andrés et al., 2006	0.004 (0.038)	0.031 (0.061)	0.027	305	0.072	0.260	[−0.023, ∞]	1.920
Parmentier and Andrés, 2010	−0.001 (0.031)	0.027 (0.056)	0.028	265	0.040*	0.325	[0.033, ∞]	3.023*
Leiva et al., 2015b	4.863 × 10^–4^ (0.045)	4.343 × 10^–4^ (0.043)	−5.2 × 10^–5^	255	0.385	0.054	[−0.232, ∞]	0.328
Leiva et al., 2016	−0.007 (0.017)	0.012 (0.040)	0.019	889	0.024*	0.263	[0.048, ∞]	4.681*
PMD_RT_								
Andrés et al., 2006	0.036 (0.035)	0.083 (0.067)	0.047	357	0.003*	0.475	[0.223, ∞]	18.116*
Parmentier and Andrés, 2010	0.028 (0.033)	0.080 (0.056)	0.052	326	< 0.001*	0.630	[0.411, ∞]	33.999*
Leiva et al., 2015b	0.022 (0.044)	0.059 (0.058)	0.037	331	0.018*	0.368	[0.096, ∞]	3.601*
Leiva et al., 2016	0.030 (0.039)	0.054 (0.047)	0.024	931	0.008*	0.322	[0.112, ∞]	3.455*

B			

Models	*BF*_10_	Error %	Best M/M
PMD_PC_			
Age Group	**25.149**	1.420E-07	Best
Study + Age Group	7.371	1.416	0.293
Study + Age Group + Study × Age Group	1.243	1.939	0.049
Study	0.346	1.636E-05	0.014
PMD_RT_			
Age Group	**236700**	<0.001	Best
Study + Age Group	48004.861	1.405	0.203
Study + Age Group + Study × Age Group	7308.905	1.930	<0.001
Study	0.236	0.236	<0.001

*BF values for the best model are high-lighted in bold.*

### Bayesian Estimation of Age-Related Difference in Deviance Distraction

Bayesian estimation was carried out on PMD_*RT*_ and PMD_*PC*_ to generate credible posterior parameters given our data. All Gelman-Rubin diagnostic values were equal to 1 (confirming that convergence was reached), and the effective sample sizes varied between 18,579 and 62,535 (i.e., superior to 10,000, the recommended value for accurate and stable estimates of the 95% HDI bounds, Kruschke, 2015).

We report the complete set of results for every parameter in Table 3 and focus here on the key relevant findings. For PMD_*RT*_, the point estimate for the mean difference between young and older adults was 0.035 (95% HDI: 0.022–0.076), indicating that unexpected sounds produce a proportional increase of RT that is 3.5% greater in older than in young adults. The point estimate of the mean effect size for this difference was 0.8 (95% HDI: 0.496–1.114), a large effect (Cohen, 1988; Lakens, 2013). Of importance, neither of the 95% HDIs included the zero value. It is worth pointing out that this 95% HDI does not overlap with a Region Of Practical Equivalence (ROPE) corresponding to a small effect size (*d* = 0.2) centered around zero (Kruschke, 2011, 2018). Hence, we can be confident that the effect of aging on PMD_*RT*_ is not equivalent to a null or a small effect.

**TABLE 3:** Posterior parameters of the Bayesian Estimation of the effect of aging on distraction.

Posterior parameter	Mean	95% HDI lower	95% HDI upper
PMD_PC_			
μ_Older_	0.005	−0.002	0.012
μ_Young_	−0.005	−0.010	0.000
σ_Older_	0.026	0.019	0.034
σ_Young_	0.022	0.017	0.027
**Older** - **Young**			
μ	0.010	0.002	0.019
σ	0.004	−0.003	0.012
Normality (log_10_*ν*)	0.441	0.240	0.643
Effect size	0.419	0.074	0.760
PMD_RT_			
μ_Older_	0.065	0.053	0.076
μ_Young_	0.029	0.022	0.037
σ_Older_	0.051	0.040	0.062
σ_Young_	0.036	0.030	0.042
**Older** - **Young**			
μ	0.035	0.022	0.048
σ	0.015	0.004	0.026
Normality (log_10_*ν*)	1.183	0.621	1.837
Effect size	0.800	0.496	1.114

The analysis of PMD_*PC*_ revealed results in the same direction as PMD_*RT*_. The point estimate for the mean difference between the two age groups was 0.010, suggesting that, relative to their performance in the standard condition, older adults see their PC decrease by 1% more than young adults (95% HDI: 0.002–0.019). While this difference is small in absolute terms, its statistical effect size estimate was small to medium (0.419). The percentage overlap between the 95% HDI (0.074–0.760) and ROPE (as defined as above) was 3.214, suggesting that the probability that the effect size estimate is equivalent to a small effect is 0.032.

### Supplementary Analysis: Effect of Aging on Cross-Modal vs. Uni-Modal Deviance Distraction

The evidence above shows that aging increases deviance distraction in cross-modal tasks. As we pointed out earlier, past work using uni-modal auditory tasks contrasts with cross-modal studies in reporting no effect of age on deviance distraction. However, these conclusions were based on absolute differences in RT and did not consider age-related variations in response speed. Hence, as a supplementary analysis, we sought to confirm the selective effect of aging on deviance distraction by using PMD_*RT*_ to compare cross-modal and uni-modal (auditory) tasks. We included data from three of our cross-modal oddball studies (Andrés et al., 2006; Parmentier and Andrés, 2010; Leiva et al., 2016) and two past studies using an auditory uni-modal task (Leiva et al., 2015a, b). The uni-modal tasks of Leiva et al. (2015b) was identical to its cross-modal version but involved the parity categorization of an auditory (as opposed to a visual) digit. Note that because the study by Leiva et al. (2015b) compared cross-modal and uni-modal tasks within-participants, we could not include both in our analysis and opted to include the uni-modal data set. From the study by Leiva et al. (2015a), we used the data from the auditory task in which 84 participants (42 young adults, *M*_*age*_ = 20.9, and 42 older adults, *M*_*age*_ = 62.8) performed a binary tone duration judgment task in which 88% of trials involved a standard 1,000 Hz tone, and the remaining 12% involved a deviant tone. The sample included in our analysis comprised of 150 young and 138 older adults. We conducted a 2 (Age group) × 2 (task type: cross- vs. uni-modal) BANOVA on PMD_*RT*_. The results revealed strong evidence for the main effects of age group (*BF*_10_ = 12.087) and task type (*BF*_10_ = 13.927). More importantly, extreme support was found for the interaction between these factors (*BF*_10_ = 2454.720). Bayesian Mann-Whitney tests were carried out to analyze this interaction (under the hypothesis of greater distraction in older than in young adults). In line with the results reported in the sections above, older adults exhibited greater PMD_*RT*_ than young adults (*W* = 4,601, *BF*_10_ = 1127.591) in cross-modal tasks. In contrast, we found strong support for the absence of a difference between the two age groups in the uni-modal tasks (*W* = 1,714, *BF*_10_ = 0.090).

## Discussion

Past research suggested that older adults exhibit greater distraction by unexpected sounds than young adults in visual tasks, an effect particularly salient in RT (Andrés et al., 2006; Parmentier and Andrés, 2010; Leiva et al., 2015b, 2016). However, these studies did not control for age-related variations in response speed as a potential explanatory factor (Shimamura, 1994; Salthouse and Meinz, 1995; Salthouse, 1996). We addressed that issue by revisiting these studies using a PMD. The results from the analysis of RT were unambiguous: older adults exhibited greater deviance distraction than young adults when controlling for baseline response speed (in each individual study and in the combined data set). Bayesian estimation revealed a proportional lengthening of RT by unexpected sounds that was about twice as large in older than in young adults (0.069 vs. 0.029, respectively, a statistically large effect). The analysis of the PC produced similar results, though the effect was statistically smaller overall and somewhat variable across studies. Overall, for both dependent measures, aging increased distraction, thereby ruling out a speed-accuracy trade-off. Finally, an additional analysis confirmed the selective effect of aging on distraction in cross-modal as opposed to uni-modal tasks.

The results summarized above bolster the view that aging is associated with a greater sensitivity to distraction by unexpected sounds when participants are performing a visual task. This contrasts with the absence of age-related difference in uni-modal oddball studies (Mager et al., 2005; Horváth et al., 2009; Getzmann et al., 2013; Leiva et al., 2015a, b). In these studies, unexpected sounds yielded distraction to similar levels in young and older adults. While the two sets of studies yield divergent findings regarding the impact of aging on distraction, they converge in dismissing general slowing as a key variable. Indeed, uni-modal oddball tasks generated similar results despite diverging with respect to overall age-related differences in response latencies: Two studies observed overall slower responses in older than in young adults (Leiva et al., 2015b), while three did not (Mager et al., 2005; Horváth et al., 2009; Getzmann et al., 2013). Though difficult to pinpoint the factor(s) underpinning this discrepancy, it may relate to the more demanding nature of the task used in the first two (parity judgment applied to a set of six possible stimuli) compared to the other three (short/long duration judgment). In any case, the fact that all five studies reported equivalent levels of distraction in the two age groups strongly suggests that distraction was not a function of baseline response speed. Hence, our results and the findings from uni-modal studies converge in distinguishing deviance distraction from age-related slowing.

One possible explanation for the selective effect of aging on distraction observed in cross-modal tasks may relate to the nature of the attentional shift entailed by unexpected sounds. Parmentier et al. (2008) suggested that deviance distraction may in part originate from a shift of attention between sensory modalities. That is, the unexpected sound, by virtue of violating sensory predictions, triggers a shift of attention from the visual modality to the auditory modality, and back to the visual modality when the target stimulus is presented. The notion of a time penalty associated with cross-modal attention shifts is consistent with evidence that shifting attention between two stimuli takes longer when these are presented in distinct sensory modalities vs. in the same modality (Turatto et al., 2002, 2004; Shomstein and Yantis, 2004; Rodway, 2005; Miles et al., 2011). Such findings support the reasonable assumption that cross-modal shifts of attention may summon greater control than within-modality shifts and invoke specific higher-levels mechanisms. Furthermore, evidence also indicates that while within-modality shifts appear to mostly mobilize primary sensory cortices, cross-modal shifts involve more extensive frontal networks (Eimer et al., 2001; Talsma et al., 2008; Salmi et al., 2009). For example, some authors have proposed that frontal alpha oscillations reflect the origin of intersensory re-orienting (Misselhorn et al., 2019). Generally, this view fits well with the hierarchical organization of control in the prefrontal cortex (Koechlin et al., 2003; Koechlin and Summerfield, 2007). Of interest, aging is associated with a proportionally greater neural deterioration in prefrontal regions (West, 1996; Raz, 2000) while posterior attentional networks are relatively spared (Greenwood et al., 1993; Hartley, 1993). Hence, we posit that the locus of the age difference in distraction by unexpected sounds in visual oddball tasks may lie in the selective alteration of frontal networks underpinning the control of attention across sensory modalities. The proposition that the effect of aging unravels in relatively late stages of processing, after the initial detection of change, fits well with EEG findings indicating that aging does not modulate early markers of the orienting response in active tasks (Berti et al., 2017).

## Conclusion

In conclusion, our study provided evidence that distraction by unexpected sounds in visual tasks is greater in older than in young adults, and that this effect cannot be accounted by variations in baseline performance between the two age groups. The results of Bayesian estimation indicate that the proportional lengthening of RT following an unexpected sound is about twice as large for older than for young adults. Interpreted in a wider context, our results point to differences in the mechanisms underpinning cross-modal shifts of attention as the possible locus of the age-related effect.

## Data Availability Statement

Publicly available datasets were analyzed in this study. This data can be found here: https://osf.io/78vk5.

## Ethics Statement

The studies involving human participants were reviewed and approved by the Balearic Islands Bioethics Review Board. The patients/participants provided their written informed consent to participate in this study.

## Author Contributions

AL carried out the data collection. FP performed the statistical analysis. FP wrote the first draft of the manuscript, with contributions from AL and PA. All authors contributed to the conception and design of this study, and approved the submitted version.

## Conflict of Interest

The authors declare that the research was conducted in the absence of any commercial or financial relationships that could be construed as a potential conflict of interest.

## Publisher’s Note

All claims expressed in this article are solely those of the authors and do not necessarily represent those of their affiliated organizations, or those of the publisher, the editors and the reviewers. Any product that may be evaluated in this article, or claim that may be made by its manufacturer, is not guaranteed or endorsed by the publisher.
